# Development of real-time PCR and droplet digital PCR based marker for the detection of *Tilletia caries* inciting common bunt of wheat

**DOI:** 10.3389/fpls.2022.1031611

**Published:** 2022-11-25

**Authors:** Zhaoyu Ren, Rongzhen Chen, Ghulam Muhae-Ud-Din, Mingke Fang, Tianya Li, Yazheng Yang, Wanquan Chen, Li Gao

**Affiliations:** ^1^ State Key Laboratory for Biology of Plant Disease and Insect Pests, Institute of Plant Protection, Chinese Academy of Agricultural Sciences, Beijing, China; ^2^ Department of Plant Protection, Shenyang Agricultural University, Liaoning, China; ^3^ College of Life Sciences, Yangtze University, Jingzhou, China

**Keywords:** droplet digital PCR, SCAR marker, qRT-PCR, ISSR, *Tilletia caries*, wheat common bunt

## Abstract

This is the first study reporting droplet digital PCR and quantitative real time PCR for detection of *Tilletia caries* (syn. *T. tritici*), which causes common bunt of wheat and leads to yield losses of 80% in many wheat growing areas worldwide. To establish an accurate, rapid and quantifiable detection method, we tested 100 inter simple sequence repeats (ISSR) primers and obtained a species-specific fragment (515 bp) generated by ISSR 827. Then, a specific 266 bp band for the sequence characterized amplified region (SCAR) marker was produced from *T. caries*. The detection limit reached 50 pg/μL. Based on the SCAR marker, we further developed a higher sensitivity of quantitative real time-polymerase chain reaction (qRT-PCR) with a detection limit of 2.4 fg/μL, and droplet digital PCR (ddPCR) with a detection limit of 0.24 fg/μL. Both methods greatly improved the detection sensitivity of *T. caries*, which will be contribute a lot for quickly and accurately detection of *T. caries*, which causes wheat common bunt.

## Introduction

*Tilletia caries* (syn. *T. tritici*) causes common bunt of wheat (*Triticum aestivum* L.), which is a seed-borne disease (Menzies et al., 2006; Goates, 2012) that appears in wheat-growing areas worldwide (Albughobeish, 2015). A typical symptom of the disease is that wheat kernel turns into millions of teliospores generated by the pathogen; the grains are then referred to as “bunt balls” (Mourad et al., 2018). The teliospore-released trimethylamine, which emits a fishy smell, seriously affecting the taste and quality of wheat flour (Gaudet et al., 2007). The teliospores of *T. caries* is very similar to *T. laevis*, and *T. controversa*, especially *T. controversa* is characterized as a quarantine pathogen (Peterson et al., 2009; Bishnoi et al., 2020). Therefore, accurate, and efficient detection methods to differentiate *T. caries* and *T. controversa* are important regarding management practices, and global trade of cereals. The traditional methods for identification are mainly based on the characterization of teliospores and germination features (5°C at 40 days and 15°C at 10 days for *T. controversa* and *T. caries*, respectively) (Boyd and Carris, 1998). However, it is very difficult to differentiate both pathogens based on the characterization of teliospores, both the measurements of teliospores and its reticulum of *T. caries* and *T. controversa* have big overlap. Overall, both methods mentioned above were laborious, time-cost and unable to quickly meet the demand for diagnosis, especially for the plant quarantine pathogen *T. controversa*. In previous studies, some methods based on internal transcription spacers (ITSs), specific DNA fragments, and DNA molecular diagnosis technology were used for the identification of different *Tilletia* spp. (Kochanová et al., 2004; Eibel et al., 2005; Carris et al., 2006), but all these methods have low sensitivity to identify *T. caries* successfully. Thus, it is of great significance and essential to develop a rapid, effective and high sensitivity detection method for *T. caries*.

With the development of molecular diagnosis technology, PCR has been widely used to differentiate *Tilletia* spp. Kochanová et al. (Kochanová et al., 2004) developed a PCR detection method for *T. caries* and *T. controversa*. The repetitive extragenic palindromic PCR (Rep-PCR) fingerprinting technique used for the identification of *Tilletia* spp. (McDonald et al., 2000; Župunski et al., 2011). Nian et al. (Nian et al., 2007) developed multiple PCRs to distinguish *T. controversa* from *T. caries*, and the lowest detectable concentration was 10 fg/μL. The qRT-PCR is characterized by its multiplexing capacity and wide range of detection with high sensitivity (Lillsunde Larsson and Helenius, 2017), but it has also been criticized for inevitably producing false-positives (Suo et al., 2020). Moreover, the results of qRT-PCR cannot be easily analyzed without a standard reference curve. The ddPCR have been reported to be used to detect a variety of plant pathogens, such as *Aspergillus flavus* (Schamann et al., 2022), *Fusarium oxysporum* f. sp.*vasinfectum* (Davis et al., 2022), *Monilinia fructicola and Monilinia laxa* (Raguseo et al., 2021), *T. controversa* (Liu et al., 2020), and *T. laevis* (Xu et al., 2020) which demonstrates the practicability and potential of ddPCR detection methods, especially in the context of a small number of target organisms. The ddPCR can still play an important role because of its sensitivity. Due to the dilution and distribution of samples across many reaction droplets, ddPCR results are more accurate and reliable than those obtained with other methods. Target DNA could be detected by a specific fluorescent labeling probe at a relatively low concentration (Hindson et al., 2011). Similarly, ddPCR technology has been applied for the identification of *T. controversa* and *T. laevis*, with detection limits of 2.1 copies/μL and 1.5 copies/μL, respectively (Liu et al., 2020; Xu et al., 2020).

In this study, we successfully developed the sequence characterized amplified region (SCAR) marker method for the identification of *T. caries*, and based on the SCAR marker, qRT-PCR and ddPCR detection methods were also successfully developed with high sensitivity. This is the first report on the detection of teliospores of *T. caries* by qRT-PCR and ddPCR method which based on SCAR marker from ISSR.

## Materials and methods

### Fungal isolates and DNA extraction

In this study, five wheat pathogens (Blumeria graminis, F. graminearum, Puccinia graminis var. tritici, P. striiformis and P. triticina) and six Tilletia-related fungi (Ustilago tritici, U. hordei, U. maydis, T. laevis and T. controversa, T. caries) were used to detect the specificity. Information on the pathogens listed in **Table S1**. The DNA of all the strains was extracted by the CTAB method (Xu et al., 2020). Then, a NanoDrop 3300 fluorospectrometer (Biotech, USA) was used to evaluate the purity and quantitation of DNA with an OD_260_/OD_280_ between 1.8 and 2.0. The integrity of the DNA was examined by agarose gel electrophoresis with λDNA/HindIII labeling. Finally, the DNA were stored at −20°C for further use.

### ISSR-PCR amplification

To obtain the species-specific DNA fragment, 100 ISSR primers published by the University of British Columbia (https://www.michaelsmith.ubc.ca/services/NAPS/PrimerSets) were used to amplify the DNA of all the tested pathogens. The primers were synthesized by Tsingke Biotechnology Co., Ltd. (Beijing, China), and are listed in **Table 1**. ISSR-PCR was performed in a programmable optics module thermocycler (Bio-Rad, USA) with a total reaction volume of 25 μL, which included 2 μL of primer (10 μM), 1 μL of template DNA (100 ng/μL), 12.5 μL of 2 × Pro Taq Master Mix (dye plus) (Vazyme Biotech Co., Ltd., China), and 9.5 μL of ddH_2_O. The PCR amplification programs were as follows: predenaturation at 94°C for 30 s; followed by 35 cycles of denaturation at 98°C for 10 s, annealing at 45–65°C (depending on the primers) for 30 s, and extension at 72°C for 1 min; and final extension step at 72°C for 2 min. The PCR products were electrophoresed on 2% agarose gel electrophoresis containing ethidium bromide at 150 V for 20 min and visualized by a gel documentation system (WSE-5200 Printgraph 2 M, ATTO, Korea).

**Table 1:** Primers used in the study.

Name	Primer sequences
M13F M13R ISSR827	5’-GTTTTCCCAGTCACGAC-3’ 5’-CAGGAAACAGCTATGAC-3’ 5´- ACA CAC ACA CAC ACA CG -3´
Erc 19F Erc 19R	5’-CTTGTCCAAGCACGTACC-3’ 5’-CTGCGCAGCGAGAGTAG-3’
Qerc19F Qerc19R	5’-GCTTTCTGTTGTTTGCTGTTGA-3’ 5’-ATCGGCTGGCTGATGTCTATA-3’
WX-F WX-R Probe primer WX-P	5’-AGGAGTCAGTAGTCAGTAGTCAG-3’ 5’-GGGAGTCGGTGGTGTAATTT-3’ FAM 5’-CTTTGGCCGTGGTGGATACCTATAGC-3’ TAMRA

### Cloning species-specific DNA fragment and SCAR marker development

The DNA fragment (515 bp) specific to *T. caries* produced by the ISSR 827 primer was isolated from the gel and purified with a TIANgel Purification Kit (TianGen Biochemical Technology Co., Ltd., China). Then, a *pCloone007* Versatile Simple Vector Kit (Tsingke Biotechnology Co., Ltd., China) was used to ligate the specific DNA fragment with the *pUC19* vector and transform it into chemically competent *Escherichia coli* DH5α cells (Tsingke Biotechnology Co., Ltd.). The cloned fragment was sequenced with primers M13F and M13R by Tsingke Biotechnology Co., Ltd. The *E. coli* plasmid was then extracted by a TIANprep Mini Plasmid Kit (TianGen Biochemical Technology Co., Ltd., China), and the concentration calculated by the NanoDrop 3300 fluorospectrometer (Biotech, USA) was 240 ng/μL. Based on the sequencing results, SCAR marker primers Erc19F and Erc19R were designed by Primer Premier 6 and synthesized by Tsingke Biotechnology Co., Ltd. (Beijing, China).

### Specificity of the SCAR marker

Five wheat pathogens and five fungi closely related to *T. caries* were used as controls in this study. SCAR-based PCR amplification was carried out with a total volume of 25 μL, including 1 μL DNA template (100 ng/μL), 1 μL SCAR primer Erc19F (10 μM), 1 μL SCAR primer Erc19R (10 μM), 12.5 μL of 2 × Pro Taq Master Mix II (dye plus) (Vazyme Biotech Co., Ltd., China) and 9.5 μL ddH_2_O. The PCR amplification programs were as follows: predenaturation at 94°C for 30 s; followed by 35 cycles of denaturation at 98°C for 10 s, annealing at 61.5°C for 30 s, and extension at 72°C for 1 min; and final extension step at 72°C for 2 min. The PCR products were electrophoresed as mentioned above.

### Sensitivity of the SCAR marker

A series of diluted DNA concentrations (100 ng/μL, 50 ng/μL, 25 ng/μL, 10 ng/μL, 5 ng/μL, 1 ng/μL, 0.5 g/μL, 0.1 ng/μL, 50 pg/μL, 25 pg/μL, 10 pg/μL and 1 pg/μL) were used as templates for sensitivity. The amplification system, programs and agarose gel electrophoresis conditions were consistent with those mentioned above.

### Specificity and sensitivity of quantitative real time PCR detection method

According to the specific DNA fragment generated by ISSR 827, qRT-PCR primers Qerc19F and Qerc19R were designed and synthesized by Tsingke Biotechnology Co., Ltd. (Beijing, China). To verify the specificity of the primers, we used 10 sets of *T. laevis* DNA (100 ng/μL) and 3 sets of *T. caries* DNA (100 ng/μL) to perform qPCR. The reaction was carried out by using an ABI 7500 real-time PCR system (Applied Biosystems, Carlsbad, CA, USA) machine. The reaction was performed in 20 μL, including 1 μL DNA, 0.4 μL Qerc19F (10 μM), 0.4 μL of Qerc19R (10 μM), 8.2 μL of nuclease-free water (TransGen Biotech, China) and 10 μl of 2 × TransStart Top Green qPCR SuperMix (+DyeI/+DyeII) (TransGen Biotech, China). The program settings were as follows: 95°C for 5 min, followed by 40 cycles of 95°C for 10 s and 58°C for 30 s. Then, a series of 10-fold diluted plasmids (2.4 pg-0.24 fg) were used as templates. Three repeats for every concentration and 2 μL of nuclease-free water (TransGen Biotech, China) were used as controls for each repeat. The reaction system was the same as mentioned above.

### DdPCR detection method

Based on the success of the SCAR marker and qRT-PCR for the detection of *T. caries*, we successfully developed ddPCR detection method. Pairs of the primers WX-F and WX-R and probe primer WX-P were synthesized by Tsingke Biotechnology Co., Ltd. (Beijing, China). The probe primer WX-FRP was generated by mixing WX-F, WX-R and WX-P at a ratio of 1:1:0.5 (10 μL of WX-F, 10 μL of WX-R, 5 μL of probe primer WX-P and 225 μL of ddH_2_O). The reaction was performed in 20 µL, containing, 10 μL Prenix EX Taq (Probe qPCR) (Takara, Japan), 4 μL WX-FRP, 4 μL ddH_2_O and 2 μL DNA template (240 ng/μL), was mixed with 35 μL of droplet-generating oil (186-3005, Bio-Rad, USA) and moved to a droplet-generating card (186-4007, Bio-Rad, USA) to generate droplets in a droplet generator (QX200, Bio-Rad, USA). The products were transferred to a 96-well PCR plate (Eppendorf, Germany), and ddPCR was performed in a C1000 touch thermal cycler (Bio-Rad, USA) with the following program: predenaturation at 95°C for 5 min, 40 cycles of denaturing at 95°C for 30 s, followed by 60 s of annealing at 58°C, and extension at 98°C for 10 min. The products were diluted 10-fold in 10 gradients and transferred to a droplet reader (QX200, Bio-Rad, Hercules, CA, US). Quanta Soft (Version, 1.7.4, Bio-Rad, provided with the ddPCR system) analysis was used to generate data, and the florescence amplitude threshold was set by the JavaScript program “dedinetherain” (Jones et al., 2014) to improve the accuracy and credibility of the results to follow the previous studies (Kubista et al., 2006; Jones et al., 2014). In this experiment, we tested the *T. caries* samples with plasmid concentrations ranging from 2.4 pg/μL to 0.24 fg/μL in 3 replicates per sample, 7 repeats of *T. controversa* and *T. laevis*, and 2 repeats of ddH_2_O as negative controls.

## Results

### Specific ISSR marker screening and SCAR marker development

**Figure 1:**
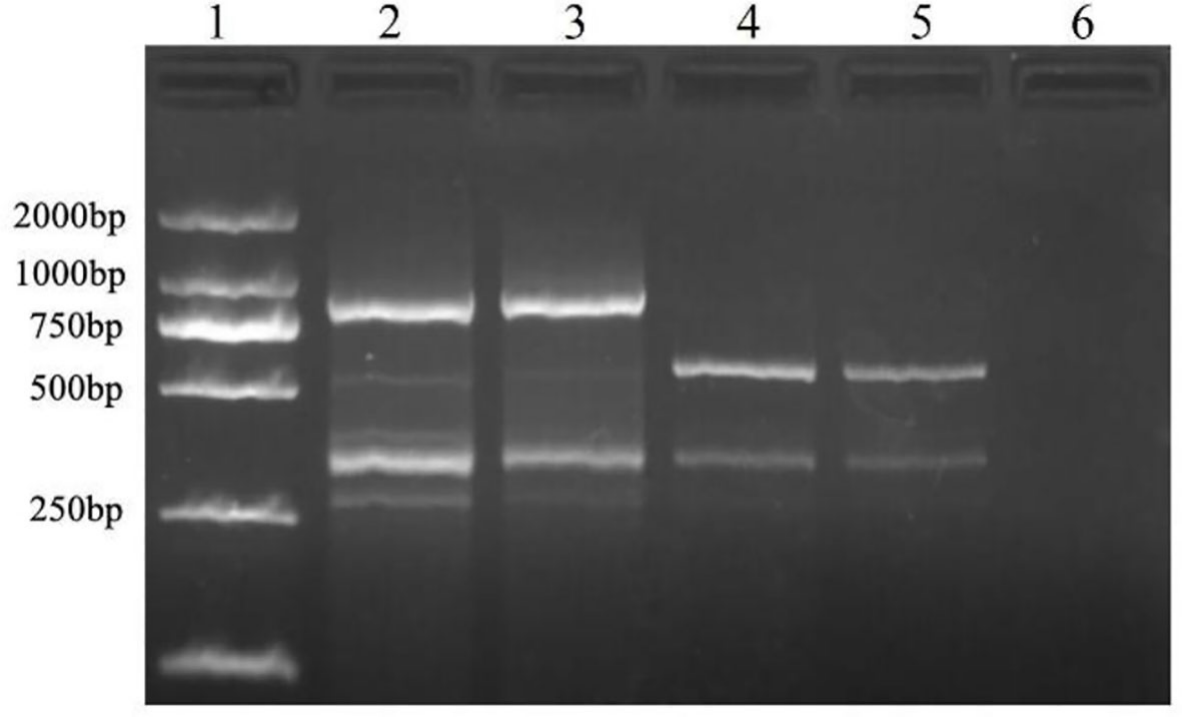
Specific fragment of *T. caries* obtained with an inter simple sequence repeat primer (ISSR827). Lane 1: DL2000 DNA ladder; lane 2: *T. controversa*; lane 3: *T. laevis*; lanes 4-5: *T. caries*; and lane 6: ddH_2_O.

**Figure 2:**
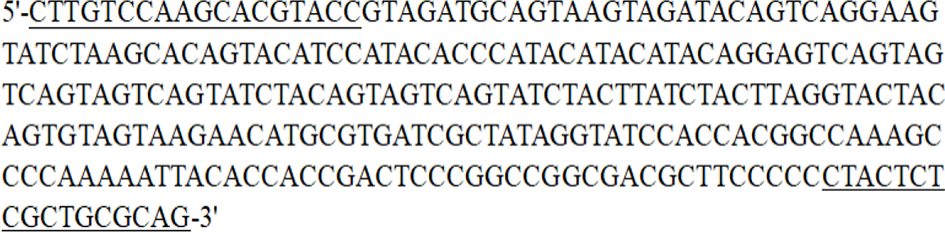
Sequence of the SCAR marker produced by Erc 19F and Erc 19R. The sequences under line are the amplification primers (Erc 19F and Erc 19R).

Among the 100 ISSR primers from the University of British Columbia, ISSR 827 amplified a polymorphic profile (515 bp) in *T. caries* but not in the other investigated pathogens (**Figure 1**). Primer Premier 6 was used to analyze the specific DNA sequence of *T. caries*, and pairs of SCAR marker primers named Erc 19F and Erc 19R were designed. The primers amplified a specific 266 bp band from the DNA of *T. caries* (**Figure 2**).

### Specificity and detection limit of the SCAR marker

**Figure 3:**
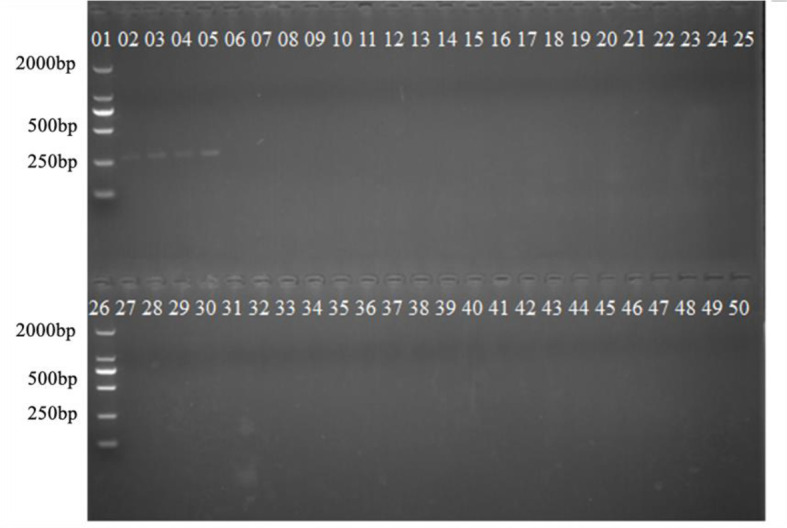
Specificity of the SCAR marker on *T. caries*. Lane 1: DL2000 DNA ladder; lanes 2–5: *T. caries;* lanes 6–9: *T. controversa*; lanes 14–17: *Ustilago maydis*; lanes 18–21: *Ustilago tritici*; lanes 22–25: *Ustilago hordei*; lane 26: DL2000 DNA ladder; lanes 27–30: *Puccinia striiformis* f. sp. *tritici;* lanes 31–34: *Puccinia triticina*; lanes 35–38: *Puccinia graminis* f. sp. *tritici*; lanes 39–42: *Blumeria graminis*; lanes 43–46: *Fusarium graminearum*; and lanes 47–50: ddH_2_O.

**Figure 4:**
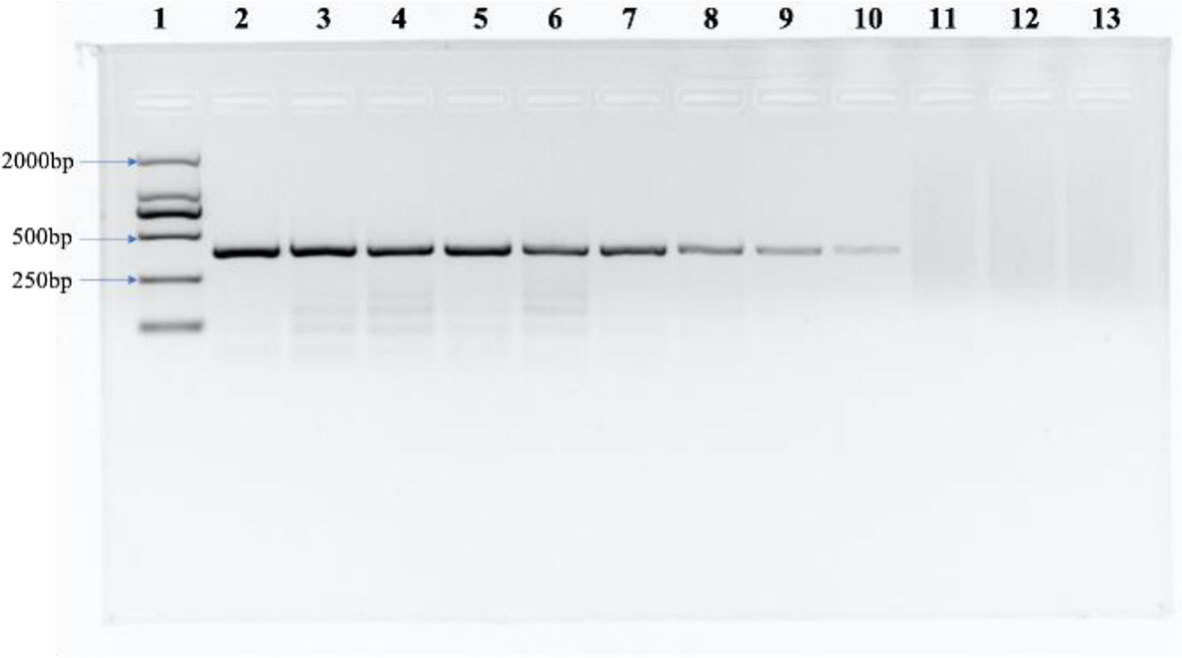
The sensitivity of the SCAR marker. Lane 1: DL2000 DNA ladder, lane 2: 100 ng/μL, lane 3: 50 ng/μL, lane 4: 25 ng/μL, lane 5: 10 ng/μL, lane 6: 5 ng/μL, lane 7: 1 ng/μL, lane 8: 0.5 ng/μL, lane 9: 0.1 ng/μL, lane 10: 50 pg/μL, lane 11: 25 pg/μL, lane 12: 10 pg/μL, and lane 13: ddH_2_O.

Some related pathogens (B. graminis, F. graminearum, P. graminis var. tritici, P. striiformis f. sp. tritici, P. triticina, U. tritici, U. hordei, U. maydis, T. caries, T. laevis and T. controversa) were used to detect the specificity of the designed SCAR primers. The specific band (266 bp) occurred only in T. caries and in none of the other tested pathogens (**Figure 3**). In addition, we tested the sensitivity of the SCAR marker using a series of diluted DNA template concentrations of T. caries (100 ng/μL, 50 ng/μL, 25 ng/μL, 10 ng/μL, 5 ng/μL, 1 ng/μL, 0.5 ng/μL, 0.1 ng/μL, 50 pg/μL, 25 pg/μL, 10 pg/μL, and 1 pg/μL), resulting in a sensitivity of 50 pg/μL of the Erc19F/Erc19R primer (**Figure 4**).

### Development of qRT- PCR detection method

**Figure 5:**
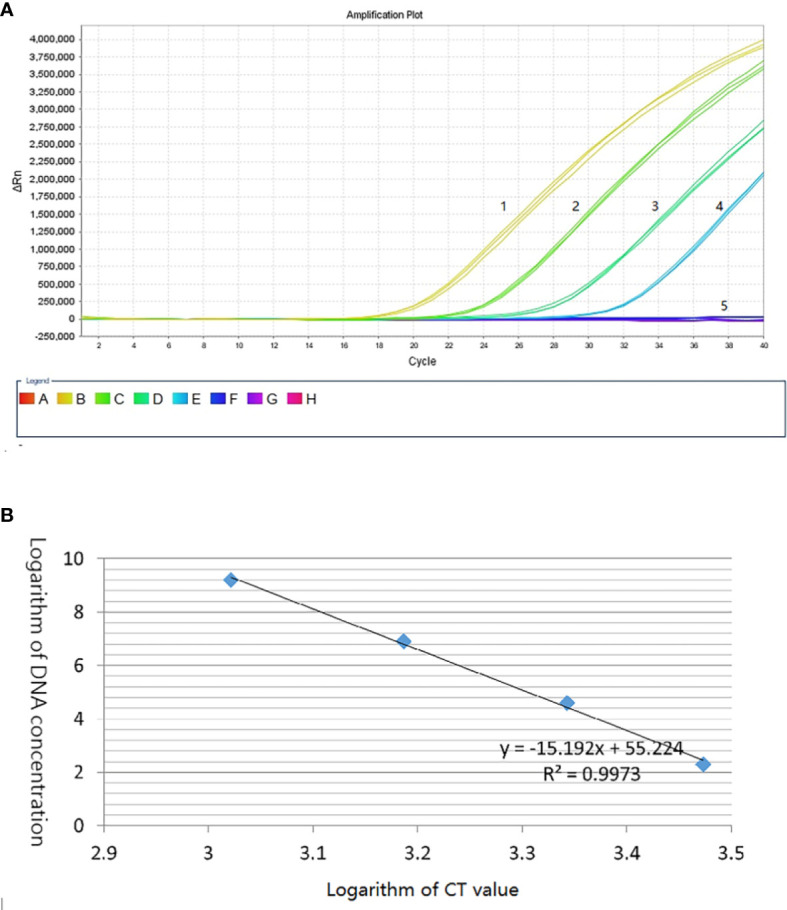
Establishment of *T. caries* standard curve by SYBR Green I real-time PCR. **(A)** Quantitative real-time amplified curves. Lanes 1–4: tenfold dilutions of recombinant plasmid DNA (2.4 pg–0.24 fg); lane 5: negative control (ddH_2_O). **(B)** Standard curve.

Based on the specific sequence, primers Qerc19F and Qerc19R were designed to perform qRT-PCR with SYBR Green I. For the specific of the primers, 10 sets of T. laevis DNA and 3 sets of T. caries DNA were used as templates to carry out qPCR. The results showed that 10 sets of T. laevis DNA failed to amplify the target sequence, while 3 sets of T. caries DNA successfully amplified the target sequence (**Supplementary Figure S1**). For the sensitivity of the detection, 10-fold serial dilutions of plasmids were used as templates (2.4 pg to 0.24 fg) and the detection limit concentration was 2.4 fg/μL (**Figure 5A**). Furthermore, a standard curve with correlation coefficient of the standard curve reached 0.997, suggesting successful development of the SYBR Green I qRT-PCR detection method (**Figure 5B**).

### The ddPCR detection method

**Figure 6:**
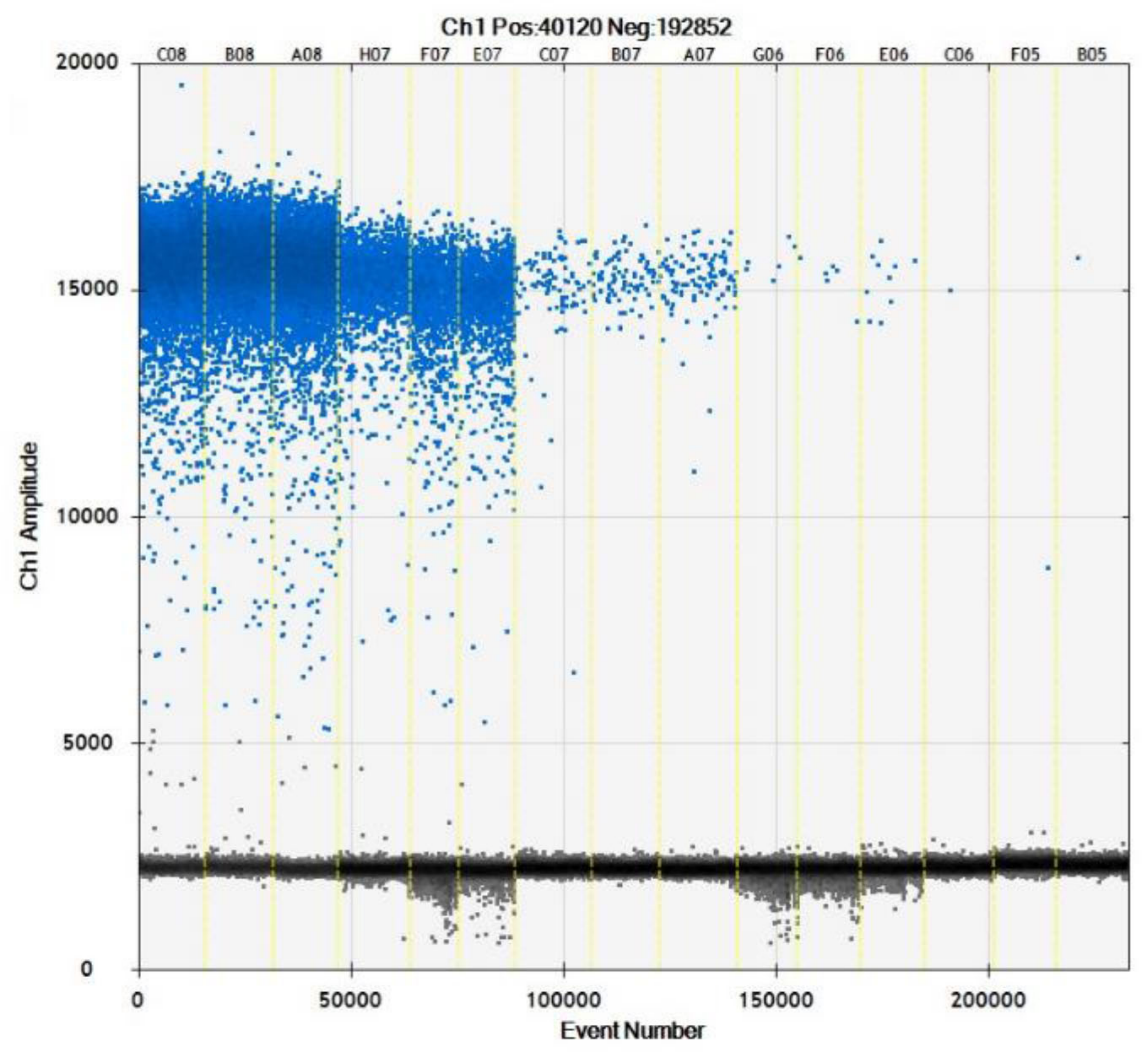
Distribution diagram of droplets of *T. caries* isolates obtained by droplet digital PCR. C08–A08, DNA template of *T. caries* (2.4 pg/μL); H07–E07, DNA template of *T. caries* (0.24 pg/μL); C07–A07, DNA template of *T. caries* (24 fg/μL); G06–E06, DNA template of *T. caries* (2.4 fg/μL), C06–B05, and DNA template of *T. caries* (0.24 fg/μL). The blue dots are positive droplets, and the black dots are negative controls.

**Figure 7:**
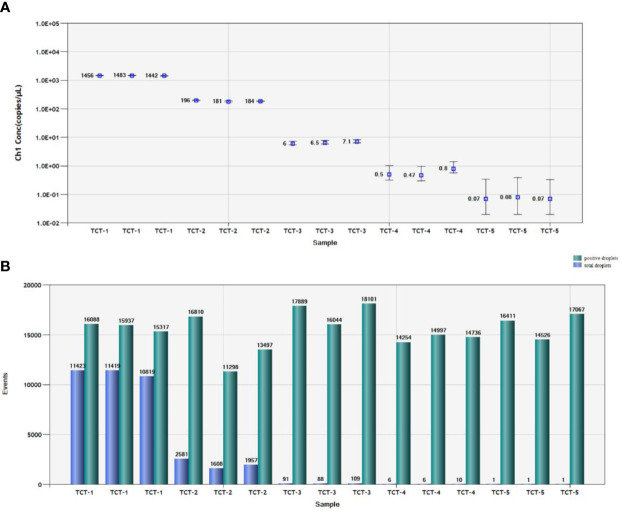
Statistical analysis by ddPCR. **(A)** Positive copy number analysis for detection of *T. caries* by copy number; TCT-1, DNA template of *T. caries* (2.4 pg/μL); TCT-2, DNA template of *T. caries* (0.24 pg/μL); TCT-3, DNA template of *T. caries* (24 fg/μL); TCT-4, DNA template of *T. caries* (2.4 fg/μL); TCT-5, DNA template of *T. caries* (0.24 fg/μL). **(B)**. Number analysis of *T. caries* isolates; TCT-1, DNA template of *T. caries* (2.4 pg/μL); TCT-2, DNA template of *T. caries* (0.24 pg/μL); TCT-3, DNA template of *T. caries* (24 fg/μL); TCT-4, DNA template of *T. caries* (2.4 fg/μL); TCT-5, DNA template of *T. caries* (0.24 fg/μL). The green pillars are the number of positive droplets, and the blue pillars indicate the number of total droplets (positive + negative).

We used 10,000 droplets to increase the accuracy and reliability of the experiment. The increase in the number of positive droplets (blue points) in the DNA samples indicated a greater copy number in the ddPCR products, which suggested that the concentration of *T. caries* in the DNA samples was increased. In contrast, the lack of positive droplets indicated that *T. caries* was not detected (**Supplementary Figure S2**). The results showed that the detection limit of ddPCR was 0.7 copies/μl (0.24 fg/μL) (**Figure 6**). Furthermore, we conducted a statistical analysis of the number of positive droplets, and the results showed that ddPCR detection is a precise and effective method for the detection of *T. caries* (**Figure 7**).

## Discussion

Until now, this is the first report on detection *T. caries* by ddPCR and qRT-PCR, even these methods were reported on *T. caries* and *T. laevis* previously (Liu et al., 2020; Xu et al., 2020). Actually, quickly detection of *T. caries* is also very important; it is difficult to differentiate *T. caries* and *T. controversa* with morphological characters. Moreover, the size and reticulum of teliospores of *T. caries* overlapped with those of *T. controversa*, a quarantine fungus of wheat in most countries.

In this research, based on the species-specific DNA band (515 bp) by ISSR 827, we developed SCAR marker (primers Erc 19F and Erc 19R). A specific band (266 bp) was amplified by the DNA of *T. caries*, but not in other related wheat pathogens, indicating the great specificity of the SCAR marker, and the detection limit of the SCAR marker was 50 pg/μL. In addition, we further developed a qRT-PCR method with a detection limit of 2.4 fg/μL, which had a higher sensitivity than the SCAR marker. The detection limit of SCAR marker and qRT-PCR was 797 and 7.97 copies/µL (1.8 and 0.018 fg/µL) for *T. controversa* respectively (Liu et al., 2020), and was 5 ng/µl and 100 fg/µl for *T. laevis* (Xu et al., 2020), while for the detection limit of our research on *T. caries*, the sensitivity of SCAR marker (50 pg/μL) and qRT-PCR (2.4 fg/μL)are higher than *T. laevis*, while lower than *T. controversa.*

The qRT-PCR can only achieve relative quantification with a standard curve, while ddPCR can achieve absolute quantification. DdPCR is more accurate and reliable than qRT-PCR (Kubista et al., 2006; Hindson et al., 2011; Pinheiro et al., 2012; Jones et al., 2014; Pavšič et al., 2016). Pieczul et al. (Pieczul et al., 2018) described the loop-mediated isothermal DNA amplification (LAMP) method for the identification of *T. caries*, *Tilletia laevis* and *T. controversa* but could not differentiate between *T. laevis*, *T. caries*, and *T. controversa*. Similarly, qPCR was used to detect the spores of *T. indica*, but could not differentiate the other bunt pathogens at the same time (Gurjar et al., 2017). However, ddPCR can differentiate *T. caries* from other bunt pathogens. Thus, we performed ddPCR for the detection of *T. caries*. The results showed that the detection limit was 0.7 copies/μL (0.24 fg/μL), which is 10-fold more sensitive than the qRT-PCR method and the detection sensitivity was higher than the previous method used for *T. controversa* (2.1 copies/μL (Liu et al., 2020) and *T. laevis* (1.5 copies/μL (Xu et al., 2020). In addition, we only found droplets with the samples of *T. caries* while not in other two similar pathogens, *T. controversa* and *T. laevis*, and can also found droplets with 0.7 copies/μL (0.24 fg/μL), which indicate the reliability of this method and reliability of the detection threshold. All the methods developed in this study showed great specificity and sensitivity and could be used as powerful tools for *T. caries* detection in the future.

## Conclusion

We successfully developed ddPCR and qRT-PCR detection method for *T. caries*, the teliospores of *T. caries* is very similar to the teliospores of *T. controversa*. Based on the results of this study, ddPCR is more sensitive than qRT-PCR based on SCAR marker. This is the first study to develop ddPCR technique and qRT-PCR detection method for *T. caries*, which caused common bunt of wheat.

## Data availability statement

The original contributions presented in the study are included in the article/**Supplementary Material**. Further inquiries can be directed to the corresponding author.

## Author contributions

Conceptualization, LG. Methodology, RC. Validation, ZR, MF, YY. Resources, WC. Writing—original draft preparation, LG and RC. Writing—review and editing, LG and GM-u-D. All authors contributed to the article and approved the submitted version.

## Funding

This research was funded by National Natural Science Foundation of China (grant numbers 31761143011 and 31571965), China Agriculture Research System (CARS-3), and Agricultural Science and Technology Innovation Program (CAAS-ASTIP).

## Conflict of interest

The authors declare that the research was conducted in the absence of any commercial or financial relationships that could be construed as a potential conflict of interest.

## Publisher’s note

All claims expressed in this article are solely those of the authors and do not necessarily represent those of their affiliated organizations, or those of the publisher, the editors and the reviewers. Any product that may be evaluated in this article, or claim that may be made by its manufacturer, is not guaranteed or endorsed by the publisher.

## Supplementary material

Supplementary Figure 1 Specificity of quantitative real time PCR detection method. Lanes 1: DNA of *T. caries*; lane 2: DNA of *T. laevis* and *T. controversa*

Supplementary Figure 2 Amplification of *T. controversa* and *T. laevis*. A02-H02, DNA template of *T. controversa*; A03-B03, ddH_2_O; A04-G04, DNA template of *T. laevis*
