# Supplementary figures and images for: Development of real-time PCR and droplet digital PCR based marker for the detection of Tilletia caries inciting common bunt of wheat
Source: Front Plant Sci. 2022 Nov 25;13:1031611. doi: 10.3389/fpls.2022.1031611 (PMC9732894; doi:10.3389/fpls.2022.1031611)

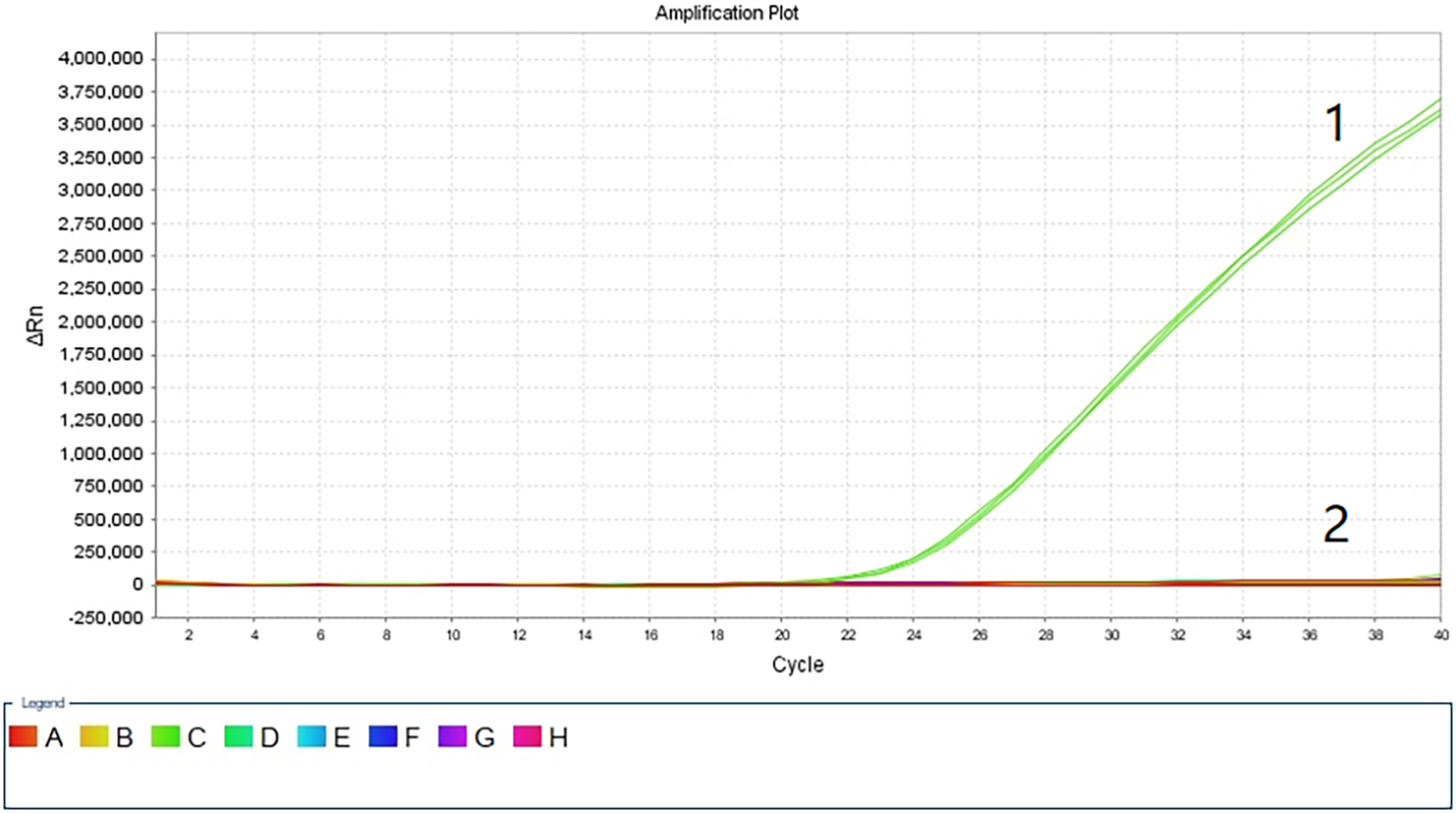

Supplement: Supplementary Figure 1 — Specificity of quantitative real time PCR detection method. Lanes 1: DNA of T. caries; lane 2: DNA of T. laevis and T. controversa [file Image_1.tiff]

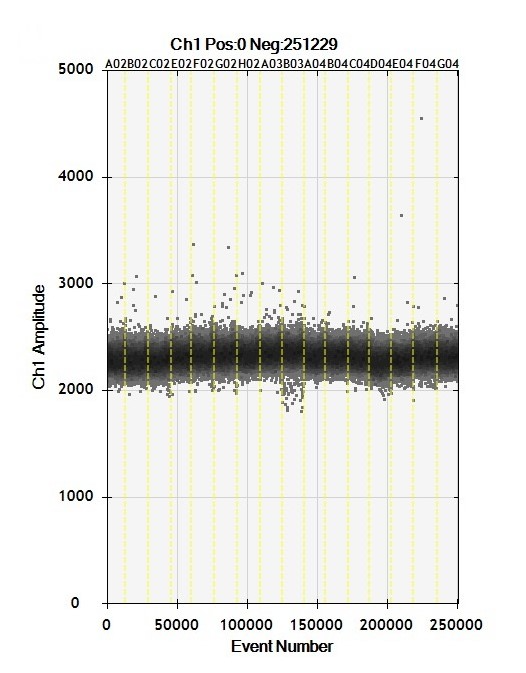

Supplement: Supplementary Figure 2 — Amplification of T. controversa and T. laevis. A02-H02, DNA template of T. controversa; A03-B03, ddH2O; A04-G04, DNA template of T. laevis [file Image_2.tiff]
