# Supplementary material for: Development of real-time PCR and droplet digital PCR based marker for the detection of Tilletia caries inciting common bunt of wheat
Source: Front Plant Sci. 2022 Nov 25;13:1031611. doi: 10.3389/fpls.2022.1031611 (PMC9732894; doi:10.3389/fpls.2022.1031611)
Supplement: Supplementary file 3 [file Table_1.docx]

**Table S1 The information of the isolates used in this study**

| **Isolates** | **Origin** | **Isolated and identified information** |
| --- | --- | --- |
| *Tilletia laevis* | Henan, China | Prof. Li Gao |
| *Tilletia controversa* | USDA-ARS | Gift from Mr Blair Goates (USDA-ARS) |
| *Tilletia caries* | Henan, China | Prof. Li Gao |
| *Ustilago tritici* | Gansu, China | Prof. Li Gao |
| *Ustilago hordei* | Qinghai, China | Prof. Li Gao |
| *Ustilago maydis* | Beijing, China | Prof. Wang Xiaoming (ICS, CAAS) |
| *Puccinia striiformis* f. sp. *tritici* | Gansu, China | Prof. Wanquan Chen |
| *Puccinia graminis* f.sp*. tritici,* | Shenyang, China | Prof. Wanquan Chen |
| *Puccinia triticina* | Hebei, China | Prof. Wanquan Chen |
| *Fusarium graminearum* | Jiangsu, China | Prof. Li Gao |
| *Blumeria graminis* | Henan, China | Prof. Li Gao |
